# Pull in and Push Out: Mechanisms of Horizontal Gene Transfer in Bacteria

**DOI:** 10.3389/fmicb.2018.02154

**Published:** 2018-09-06

**Authors:** Dongchang Sun

**Affiliations:** College of Biotechnology and Bioengineering, Zhejiang University of Technology, Hangzhou, China

**Keywords:** antibiotic resistance gene, multidrug resistance, DNA transfer, natural transformation, conjugation, *Escherichia coli*

## Abstract

Horizontal gene transfer (HGT) plays an important role in bacterial evolution. It is well accepted that DNA is pulled/pushed into recipient cells by conserved membrane-associated DNA transport systems, which allow the entry of only single-stranded DNA (ssDNA). However, recent studies have uncovered a new type of natural bacterial transformation in which double-stranded DNA (dsDNA) is taken up into the cytoplasm, thus complementing the existing methods of DNA transfer among bacteria. Regulated by the stationary-phase regulators RpoS and cAMP receptor protein (CRP), *Escherichia coli* establishes competence for natural transformation with dsDNA, which occurs in agar plates. To pass across the outer membrane, a putative channel, which may compete for the substrate with the porin OmpA, may mediate the transfer of exogenous dsDNA into the cell. To pass across the inner membrane, dsDNA may be bound to the periplasmic protein YdcS, which delivers it into the inner membrane channel formed by YdcV. The discovery of cell-to-cell contact-dependent plasmid transformation implies the presence of additional mechanism(s) of transformation. This review will summarize the current knowledge about mechanisms of HGT with an emphasis on recent progresses regarding non-canonical mechanisms of natural transformation. Fully understanding the mechanisms of HGT will provide a foundation for monitoring and controlling multidrug resistance.

## Introduction

Horizontal gene transfer (HGT) drives the evolution of bacteria. Transfer of antibiotic resistance genes (ARGs) plays an important role in the development of multidrug resistance (MDR) in bacteria (Forsberg et al., 2012). There are three “classical" methods of DNA transfer in nature: bacterial conjugation, natural transformation, and transduction (von Wintersdorff et al., 2016). Via HGT, exogenous DNA can be transferred from one bacterium to another even if they are only distantly related (Chen et al., 2005; Burton and Dubnau, 2010). With the accumulation of genes involved in different resistance mechanisms from the exogenous DNA, bacteria are able to acquire MDR rapidly. For example, *Acinetobacter* and *Enterobacter* strains carrying the NDM-1 plasmid or *mcr-1* plasmid, which contain a group of resistance genes, can tolerate even last-resort antibiotics (Yong et al., 2009; Wang and Sun, 2015; Liu et al., 2016; Shen et al., 2016; Zheng et al., 2017). Understanding the mechanisms of DNA transfer in bacteria would provide new strategies to help address the ongoing challenge of multi-drug-resistant bacteria in the future.

Natural bacterial transformation and conjugation have been found in bacteria and archaea. Two different membrane protein complexes consisting of conserved proteins are responsible for pulling in and pushing out DNA during natural bacterial transformation and conjugation, respectively (Chen and Dubnau, 2004; Claverys et al., 2009; Burton and Dubnau, 2010; Johnston et al., 2014; Cabezon et al., 2015; Ilangovan et al., 2015, 2017). These DNA transport systems deliver single-stranded DNA (ssDNA) either from the donor cell (for conjugation) or into the recipient cell (for transformation) (Chen and Dubnau, 2004; Claverys et al., 2009; Burton and Dubnau, 2010; Johnston et al., 2014; Cabezon et al., 2015; Ilangovan et al., 2015, 2017). Recent studies have revealed two new types of DNA transfer in *Escherichia coli*. One of these methods has been shown to be independent of the conserved proteins for the transport of ssDNA during natural transformation or bacterial conjugation. Instead, double-stranded DNA (dsDNA) is taken up into the cytoplasm and internalized by *E. coli* cells on solid agar plates (Sun et al., 2006, 2009, 2013; Sun, 2011, 2016; Zhang et al., 2012). The other method of DNA transfer is dependent on cell-to-cell contact and DNA transfer occurs within a colony on agar plates (Maeda et al., 2004, 2006; Etchuuya et al., 2011; Sobue et al., 2011; Kurono et al., 2012; Matsuda et al., 2012; Matsumoto et al., 2016). DNA transfer via this method is sensitive to DNase I, indicating that DNA that is transported into the recipient cell is naked (rather than protein-protected). Although DNA transfer occurs on agar plates via both of the above two transformation methods, no evidence shows similarities between DNA transfer mechanisms of the two methods. In the former transformation method, DNA transfer occurs in the absence of donor cells. Whereas, in the latter transformation method, donor cells are required for DNA transfer in the colony. During bacterial conjugation, physical contact between the donor and the recipient cells is required. Nonetheless, the cell-to-cell contact-dependent plasmid transformation is different from conjugation in that DNA transfer is not mediated by mobile elements (Maeda et al., 2004; Kurono et al., 2012; Matsuda et al., 2012). In this review, we will first discuss the mechanisms of classical natural bacterial transformation and conjugation. Then, the non-canonical DNA transfer on agar plates will be described in detail, with an emphasis on how DNA is pulled into cells.

## Pull Dna in During Natural Transformation

Natural transformation was discovered in *Streptococcus pneumoniae* in 1928 (Griffith, 1928). Induced by a heptadecapeptide pheromone, naturally transformable bacteria show a special physiological state termed “competence”, during which they are capable of pulling in exogenous DNA (Berka et al., 2002; Ogura et al., 2002). The mechanism of DNA transfer during natural transformation is well conserved among Gram-positive (G^+^) (e.g., *Bacillus subtilis* and *S. pneumoniae*) and Gram-negative (G^−^) bacterial species (e.g., *Neisseria gonorrhoeae*, *Haemophilus influenzae*, and *Vibrio cholerae*), as well as archaea (Chen and Dubnau, 2004; Claverys et al., 2009; Burton and Dubnau, 2010; Johnston et al., 2014; Cabezon et al., 2015; Ilangovan et al., 2015, 2017; Veening and Blokesch, 2017). Although conditions for competence induction vary widely among bacterial species (Aas et al., 2002; Berka et al., 2002; Claverys et al., 2006; Veening and Blokesch, 2017), proteins involved in DNA uptake are highly conserved even among distantly related bacteria (Johnston et al., 2014), except for *Helicobacter pylori*, which uses a conjugation-like system for DNA uptake during natural transformation (Smeets and Kusters, 2002). Here, the conserved DNA uptake system in bacteria is described.

**FIGURE 1:**
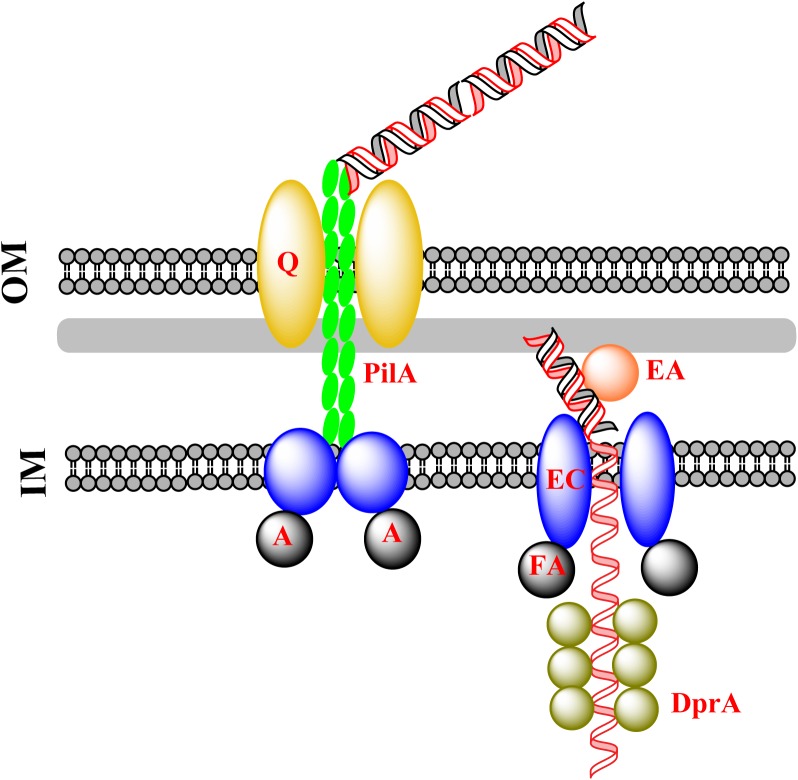
Classical DNA uptake during natural transformation. Exogenous DNA is pulled into the cytoplasm by the extension and retraction of pseudopili, as a consequence of the assembly and disassembly of pseudopilin multimers (PilA). This is followed by the transfer of dsDNA across the OM protein PilQ/HofQ (for G^−^ bacteria only). The DNA receptor (ComEA) mediates the transfer of one strand of DNA across the IM channel formed by ComEC with the assistance of the ATPase ComFA, accompanied by degradation of the other strand of DNA. The incoming ssDNA is protected by DprA which conveys it to RecA for homologous recombination in the cytoplasm.

G^−^ bacteria have an outer membrane (OM), whereas G^+^ bacteria have not. During natural transformation, G^−^ bacteria need to pull DNA across both the OM and the inner membrane (IM), whereas G^+^ bacteria need to overcome the barrier of the peptidoglycan layer, which is much thicker and denser, and needs to be weakened before translocation of DNA across the IM. In this process, dsDNA is pulled across the OM in G^−^ bacteria and ssDNA is pulled across the IM in both G^+^ and G^−^ bacteria. The general mechanism underlying DNA transfer during natural transformation is summarized in **Figure 1**.

### Crossing the OM

For G^−^ bacteria, a sophisticated protein complex is assembled in the OM, where the complex binds exogenous DNA and drags it into the periplasm (**Figure 1**). The assembly and disassembly of a type IV pilus causes a fiber-like pseudopilus to be extruded out of and hauled back into the pore-forming OM proteins (Chen and Dubnau, 2004). The pore that accommodates exogenous DNA is 6–6.5 nm in diameter, and is formed by PilQ, a secretin that is 15 nm wide and 34 nm long with five rings and an extraordinary stable “cone” and “cup” structures (Chen and Dubnau, 2004). The pore cavity is large enough to accommodate dsDNA (∼2.4 nm) (Collins et al., 2001; Assalkhou et al., 2007; Burkhardt et al., 2011). Accompanied by the extension and retraction of type IV pili, DNA is transported across the OM through the pore (Laurenceau et al., 2013; Salzer et al., 2014, 2016; Leong et al., 2017). Between the OM and IM (the periplasm), the incoming DNA is bound by the substrate-binding protein ComEA, which prevents DNA from slipping by means of a “Brownian Ratcheting” mechanism (Inamine and Dubnau, 1995; Provvedi and Dubnau, 1999; Berge et al., 2002; Takeno et al., 2012; Seitz et al., 2014; Salzer et al., 2016).

### Crossing the IM

Bound by ComEA in the periplasm, exogenous DNA is translocated across the IM via a pore formed by ComEC, also named Rec2 in some G^−^ bacteria (e.g., *H. influenzae*), a widely conserved IM protein for translocation of DNA across the IM (Barouki and Smith, 1985; Berge et al., 2002; Draskovic and Dubnau, 2005; Sinha et al., 2012; Baker et al., 2016; Salzer et al., 2016). It has been proposed that ComEC acts as both a translocase and nuclease during DNA translocation (Chen and Dubnau, 2004; Claverys et al., 2009; Burton and Dubnau, 2010; Johnston et al., 2014; Veening and Blokesch, 2017). In this process, one strand of the dsDNA is translocated into the cytoplasm, simultaneously the degradation of the other strand occurs (Barouki and Smith, 1985; Berge et al., 2002; Baker et al., 2016). *In silico* analysis of ComEC indicates that the β-lactamase-like domain at the C-terminus may function as a nuclease and Domain of Unknown function 4131 at the N-terminus has a DNA binding domain (Baker et al., 2016). It is possible that the dsDNA that is bound to the N-terminus of ComEC is divided into two strands of ssDNA and one of them is degraded by the nuclease at the C-terminus.

The channel for DNA translocation is assumed to be formed by two ComEC monomers with seven transmembrane segments (Draskovic and Dubnau, 2005). Given that over-expression of ComEC is toxic to the cell, the structure of ComEC remains unresolved (Draskovic and Dubnau, 2005). The driving force for translocation of ssDNA across the IM may be provided by an ATPase (ComFA), which is also widely conserved in bacteria (Londono-Vallejo and Dubnau, 1994; Takeno et al., 2011; Chilton et al., 2017; Diallo et al., 2017). After translocation of ssDNA, DprA, and RecA bind ssDNA and catalyze the formation of joint DNA molecules for homologous recombination (Mortier-Barriere et al., 2007; Dwivedi et al., 2013; Yadav et al., 2013, 2014; Duffin and Barber, 2016; Diallo et al., 2017; Hovland et al., 2017; Le et al., 2017). In this way, the incoming foreign ssDNA displaces one strand of the chromosomal dsDNA (Mortier-Barriere et al., 2007), followed by being converted to homogeneous dsDNA through DNA replication.

## The Transfer of Dna During Bacterial Conjugation

**FIGURE 2:**
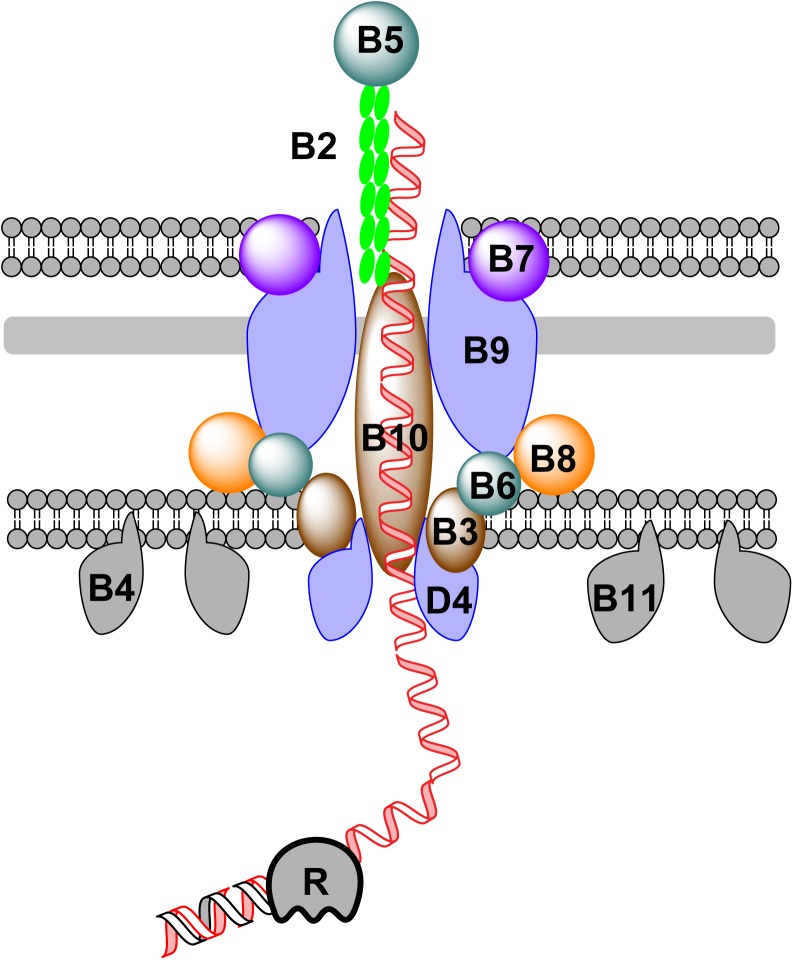
DNA transfer during bacterial conjugation. Conjugative DNA is processed into ssDNA by a relaxase (R) in the cytoplasm of the donor bacterium. To further transport ssDNA, a group of membrane and periplasmic proteins are assembled together to form a large complex, which can be subdivided into four distinct parts: (1) the pilus is formed by the assembly of pilins (VirB2) with adhesins (VirB5) at the distal end; (2) the OM component, which consists of VirB7, VirB9, and the C-terminus of VirB10; (3) the periplasmic component which consists of VirB8, VirB10, and VirB6; and (4) the IM component, formed of VirB3, VirB6, VirB8, and VirB10. Additionally, three hexameric ATPases (VirB4, VirB11, and VirD4) are attached to the IM to provide energy during DNA transfer.

Bacterial conjugation was first discovered in *E. coli* (Lederberg and Tatum, 1946). Relying on cell-to-cell contact, DNA can be pushed out of a donor cell and transported into a recipient cell during bacterial conjugation. A group of modular mobile genetic elements, known as integrative and conjugative elements (ICEs) or conjugative transposons (Franke and Clewell, 1981), has been found in many bacterial genomes (Wozniak and Waldor, 2010; Bi et al., 2012; Cury et al., 2017). ICEs can transfer from one bacterium to another, facilitating the spread of ARGs in environment (Wozniak and Waldor, 2010; Bi et al., 2012; Cury et al., 2017). The transfer of conjugative DNA across the membrane of the donor bacterium relies on a large membrane-associated protein complex, that belongs to the type IV secretion system (T4SS) (Goessweiner-Mohr et al., 2013; Cabezon et al., 2015; Ilangovan et al., 2015; **Figure 2**). Components of the T4SS for conjugation are encoded by genes of either self-replicable conjugative plasmids or ICEs in the chromosomal DNA of the donor bacterium (Cabezon et al., 2015; Ilangovan et al., 2015; Johnson and Grossman, 2015). The mechanism of DNA transfer is summarized in **Figure 2**.

Protein-coated conjugative ssDNA is transported across the IM, periplasm and OM through a membrane channel formed by a group of proteins encoded by the conjugative DNA molecule (Goessweiner-Mohr et al., 2013; Cabezon et al., 2015; Ilangovan et al., 2015). Inside the channel, the conjugative pilus (formed by pilins) is responsible for pushing ssDNA out of membrane. The mechanism of DNA transfer via conjugation has been best exemplified by the Vir system. To export DNA out of the donor cell, a conjugative plasmid encodes a complicated membrane protein complex. During conjugation, a plasmid- or ICE-encoded relaxase creates a nick in one strand of the conjugative DNA at the *oriT* site, followed by ssDNA translocation across the channel formed by components of the T4SS and replication of the remaining strand, either independently from or in concert with conjugation (Ilangovan et al., 2017). During translocation across cell membranes, three ATPases (VirD4, VirB4, and VirB11) provide the energy for DNA transport (Chen et al., 2005; Cabezon et al., 2015; Ilangovan et al., 2015). In natural transformation and conjugation, different types of pili participate in the movement of DNA. Competence pili or pseudopili mediate the transfer of dsDNA across the membrane during natural transformation, whereas conjugative pili mediates the transfer of ssDNA across the membrane during conjugation (Cabezon et al., 2015; Ilangovan et al., 2015). In both cases, the assembly/disassembly of pili drives the movement of transferring DNA. It remains unclear how conjugative DNA is further transported in the recipient cell.

## The Uptake of dsDna in *E. coli*

*E. coli* has long been thought not to be naturally transformable. In this century, the natural transformation of *E. coli* has been observed initially on nutrient-deficient agar plates and later on nutrient-rich agar plates (Tsen et al., 2002; Sun et al., 2006). Although natural plasmid transformation of *E. coli* shows single-hit kinetics, implying that dsDNA may enter the cell (Sun et al., 2009), there are basic differences between natural and chemical transformation. First, natural transformation is promoted by an increased concentration of agar, whereas chemical transformation relies on high concentrations of divalent ions (i.e., Ca^2+^, Mg^2+^, or Mn^2+^) (Sun et al., 2009). However, the stimulating effect of agar on transformation is not due to an increase in Ca^2+^, Mg^2+^, or Mn^2+^ concentration (Sun et al., 2009). It remains unclear whether osmotic pressure and/or any other biological/physical factor(s) contribute to the increase of transformation on plates with a high concentration of agar. Second, an OM protein, OmpA, plays opposite roles in natural and chemical transformation of *E. coli*: it promotes chemical transformation but suppresses natural transformation (Sun et al., 2013). Third, exponentially growing *E. coli* cells are often employed for preparing chemically competent cells with the highest efficiency, and chemical transformation occurs in a liquid, whereas the natural transformation of stationary-phase *E. coli* cells is regulated by the transcriptional regulator RpoS and the cyclic AMP (cAMP) – cAMP receptor protein (CRP), and these cells can acquire exogenous DNA exclusively on agar plates (Zhang et al., 2012; Guo et al., 2015). The functions of RpoS or the cAMP-CRP complex in the chemical transformation of *E. coli* have not been found.

**FIGURE 3:**
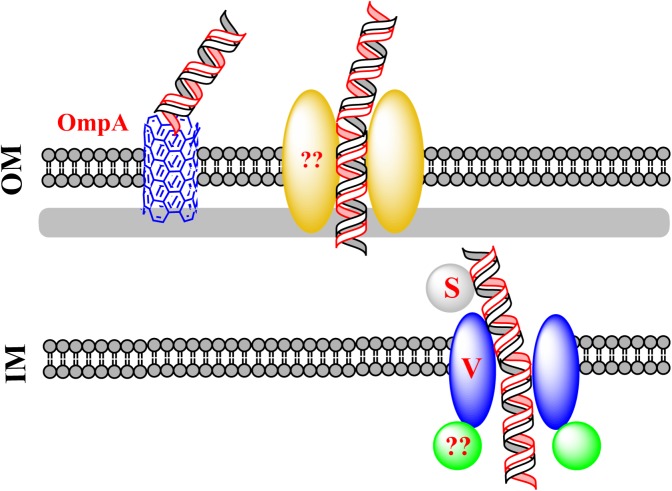
A new route for dsDNA transfer in *Escherichia coli*. Via an unidentified channel, exogenous DNA transfers across the OM. The pore-forming protein OmpA can compete for DNA with the unidentified channel. To pass across the IM, the incoming DNA binds the substrate-binding protein YdcS and is translocated from the periplasm to the cytoplasm via an IM channel formed by YdcV. It remains unclear whether a plasmid enters *E. coli* as intact circular or linear dsDNA.

Natural transformation of *E. coli* also differs from that of other naturally transformable bacteria in that the conserved DNA uptake machinery is not required for the uptake of exogenous dsDNA in *E. coli* (Sun et al., 2009). Some components of the conserved DNA uptake machinery are believed to function in using DNA as a nutrient in *E. coli* (Finkel and Kolter, 2001; Palchevskiy and Finkel, 2006). Nevertheless, attempts to confirm these observations in three independent laboratories have not succeeded (Sun, 2011; Johnston et al., 2014). DNA, which has been thought to serve as the sole carbon source could not account for cell growth, implying that other nutrient sources should be present in the culture (Sun, 2011; Johnston et al., 2014). It is possible that degraded DNA in the minimal culture serves as a source of building blocks for the synthesis of new DNA in bacteria. During the natural transformation of *E. coli*, new ABC transporter proteins have been shown to participate in DNA transfer (Sun et al., 2009; Sun, 2016). These transporters are different from the known classical DNA uptake proteins that mediate natural bacterial transformation. The mechanism of this new type of DNA transfer is proposed in **Figure 3**.

### Crossing the OM

*Escherichia coli* has a complete set of genes that potentially encode components of the classical DNA uptake machinery. These genes are homologous to the conserved DNA uptake genes in other naturally transformable bacteria. Comparative genomic analysis predicts that, in *E. coli*, putative DNA uptake genes *hofQ* and *gspD* may encode proteins forming a channel for transporting DNA across the OM, and *ppdD* may encode pilins for the assembly of the competence pili or pseudopili which pull exogenous DNA in (Finkel and Kolter, 2001; Claverys and Martin, 2003; Sun et al., 2009). However, inactivation of *hofQ*, *gspD*, or *ppdD* does not affect natural transformation with dsDNA, suggesting that the conserved DNA uptake machinery for translocation of ssDNA does not mediate dsDNA transfer across the OM in *E. coli* (Sun et al., 2009). To identify the OM pore used for DNA transport during natural transformation of *E. coli*, the pore-forming protein OmpA was evaluated, considering that OmpA performs functions in bacteriophage infection and bacterial conjugation. Inactivation of *ompA* increases natural transformation by 7- to 60-fold while decreasing chemical transformation by ∼10-fold, suggesting that OmpA blocks DNA transfer during natural transformation but promotes DNA transfer in artificial transformation (Sun et al., 2013). OmpA is unlikely to form an open gate under natural conditions, but can be switched to the open state with the molecular force of electrostatic interaction (i.e., salt-bridge), that drives structural transition of a protein under different conditions (Hong et al., 2006). The closed and open states of the gate are dependent on the formation of salt bridges of Arg138-Glu52 and Lys82-Glu128, respectively (Hong et al., 2006). During natural transformation of *E. coli*, DNA may pass across an unidentified channel that competes with OmpA for transforming DNA. The putative channel may be consisted of OM components (i.e., OM protein and pili/psedopili) of a DNA transport system and pulls DNA into the cell on the LB-agar plate. In the default “gate-closed” state, OmpA traps the transforming DNA, making it unable to reach the right channel for completing transformation. By contrast, during chemical transformation or electroporation, a high concentration of Ca^2+^ or an electric current helps open the gate, allowing DNA to pass across the channel formed by OmpA (Sun et al., 2013).

### Crossing the IM

Based on a membrane topology study, the conserved IM protein called ComEC is predicted to mediate the translocation of ssDNA during classical natural transformation (Chen and Dubnau, 2004; Claverys et al., 2009; Johnston et al., 2014). However, inactivation of the ComEC homolog YcaI in *E. coli* does not affect natural plasmid transformation of *E. coli*, suggesting that DNA is translocated into the cytoplasm via a different route (Sun et al., 2009). The single-hit kinetics in natural plasmid transformation of *E. coli* suggest that the establishment of a plasmid in the cytoplasm is mediated not by the annealing of partially overlapping opposite ssDNA derived from two independent plasmid monomers, but by a new route, i.e., the transfer of dsDNA across the IM of bacteria (Sun et al., 2009). Screening of RpoS-targeted transformation-related genes has identified *ydcS* and *ydcV*, which are located in the same ABC-transporter operon (Sun, 2016). Inactivation of *ydcS* and *ydcV* reduces natural transformation 6.7- and 9.5-fold, respectively (Sun, 2016). Chemical transformation is also reduced by Inactivation of *ydcS*, whereas the chemical transformation in a *ydcV* mutant is not reduced as compared to its wild-type counterpart (Sun, 2016). According to the Transporter Classification Database (TCDB^1^), *ydcS* and *ydcV* are predicted to encode proteins for binding a substrate in the periplasm and for translocation of the substrate across IM (Saier et al., 2014). YdcS has been shown as a PHB synthase in the periplasm (Dai and Reusch, 2008). The mutant lacking *ydcT* (a putative ATPase encoding gene) in the same operon as *ydcS* and *ydcV*, is naturally transformed with slightly but obviously reduced frequency (less than 50%) (Sun, 2016), indicating that this gene is also involved in natural plasmid transformation of *E. coli*. Nonetheless, with respect to significantly reduced transformation frequency in the *ydcV* and the *ydcS* mutants, *ydcT* seems to have only a minor effect on DNA transport. It is likely that additional energy source is required for efficient transport of dsDNA across the IM.

## Cell-To-Cell Transfer of Non-Conjugative Plasmids

Cell-to-cell contact is often required for bacterial conjugation. Of note, plasmid transformation of *E. coli* in colonies proceeds on the surface of agar plates and cell-to-cell contact is required for the transfer of plasmids that do not carry conjugative functions (Maeda et al., 2004, 2006). Cell-to-cell contact-dependent plasmid transformation occurs not only within the same genus but also across genera (Wang et al., 2007). In some naturally transformable G^−^ bacteria (e.g., *H. influenzae* and *N. gonorrhoeae*), a short DNA sequence (named DUS) can increase DNA uptake (Claverys and Martin, 2003; Wang and Sun, 2015; Zheng et al., 2017). During cell-to-cell contact-dependent plasmid transformation of *E. coli*, an 88 bp DNA sequence promotes DNA transfer (Sobue et al., 2011). Screening of the Keio collection (Baba et al., 2006), a comprehensive library of *E. coli* knock-out mutants defective in non-essential genes, has identified *rodZ*, whose product regulates the rod-shape of the cell, as an essential gene for cell-to-cell contact-dependent transformation (Kurono et al., 2012). Because the homologs of DNA uptake genes (e.g., *ycaI*) have not been revealed to be essential genes, conventional DNA uptake machinery is not likely to be involved in cell-to-cell contact-dependent plasmid transformation of *E. coli*. Recently, cell-to-cell contact-dependent transformation is discovered in *B. subtilis* (Zhang et al., 2018). However, inactivation of the competence regulator ComK, that controls the expression of conserved DNA uptake genes, abolishes cell-to-cell DNA transfer (Zhang et al., 2018), indicating that mechanisms of the cell-to-cell DNA transfer in *E. coli* and *B. subtilis* are basically different.

## Concluding Remarks

Classical mechanisms of HGT (i.e., natural transformation and conjugation) share common features in that ssDNA is pushed out or pulled into the cell with the assistance of membrane-associated protein complexes. Recent studies have uncovered new types of DNA transfer, which are independent of the classical DNA uptake or conjugation machineries, suggesting that other therapeutic targets should be considered in the fight against ARGs. The conserved proteins involved in transporting or protecting DNA may serve as targets for limiting the transfer of ARGs and in turn for reducing MDR in bacteria (Goessweiner-Mohr et al., 2013). On the other hand, the discovery of non-classical HGT mechanisms suggests that controlling the spread of ARGs is more complicated than previously thought.

## Author Contributions

DS conceived the idea and wrote the manuscript.

## Conflict of Interest Statement

The author declares that the research was conducted in the absence of any commercial or financial relationships that could be construed as a potential conflict of interest.

## Footnotes

1 http://www.tcdb.org
